# Behavioral Interventions for Tobacco Cessation in Low- and Middle-Income Countries: A Systematic Review and Meta-analysis

**DOI:** 10.1093/ntr/ntae259

**Published:** 2024-11-01

**Authors:** Abhijit Nadkarni, Leena Gaikwad, Miriam Sequeira, Pranay Javeri, Deepthy Benoy, Marimilha Grace Pacheco, Richard Velleman, Pratima Murthy, Felix Naughton

**Affiliations:** Department of Population Health, Centre for Global Mental Health, London School of Hygiene and Tropical Medicine, London, UK; Addictions and Related Research Group, Sangath, Goa, India; Addictions and Related Research Group, Sangath, Goa, India; Addictions and Related Research Group, Sangath, Goa, India; Addictions and Related Research Group, Sangath, Goa, India; Addictions and Related Research Group, Sangath, Goa, India; Addictions and Related Research Group, Sangath, Goa, India; Addictions and Related Research Group, Sangath, Goa, India; Department of Psychology, University of Bath, Bath, UK; National Institute of Mental Health & Neurosciences, Bengaluru, India; School of Health Sciences, University of East Anglia, Norwich, UK

## Abstract

**Introduction:**

An estimated 78% of the total deaths attributable to smoking tobacco use occurred in low- and middle-income countries (LMICs) in 2019. In addition, smokeless tobacco increases the risk of all-cause mortality, all cancers, including upper aero-digestive tract cancer, stomach cancer, ischemic heart disease and stroke, with 88% of the mortality burden being borne by the South-East Asian region. Evidence-based interventions from high-income countries (HICs) are not easily transferable to LMICs, as patterns of tobacco use, health beliefs associated with tobacco use, and awareness of specific health risks vary substantially.

**Methods:**

We synthesized the effectiveness of behavioral interventions for tobacco cessation in LMICs through a systematic review and meta-analysis. Interventional studies which delivered individual behavioral intervention and assessed abstinence from tobacco use were included. We examined the pooled intervention effect at 6 months postintervention follow-up.

**Results:**

For continuous abstinence at 6 months, the intervention was superior to the active comparator (RR 2.32; 95% CI 1.78 to 3.02) and usual care (RR 4.39; 95% CI 2.38 to 8.11). For point prevalence abstinence at six months, the intervention was superior to the active comparator (RR 1.76; 95% CI 1.28 to 2.44), and usual care (RR 2.37; 95% CI 1.47 to 3.81). The statistical heterogeneity was substantial to considerable for all comparisons. Only six studies had an overall low risk of bias. Publication bias was observed for all comparisons except for 6-month continuous outcomes.

**Conclusions:**

Implementation research is needed to understand factors for programme sustainability and equity of the impact of behavioral interventions in reducing tobacco use in LMICs.

**Implications:**

Our review is an important step towards understanding the effectiveness of behavior interventions for tobacco cessation suited for LMICs and which are responsive to the contextual needs of such countries.

## Introduction

In 2019, 1.14 billion current smokers consumed 7.41 trillion cigarette-equivalents of tobacco across the world.^1^ In 2020, 22.3% of the global population aged 15 years and older were current users of some form of tobacco. The global prevalence of smokeless tobacco use was estimated to be 6% among adults.^2^ Globally in 2019, smoked tobacco accounted for 7.69 million deaths and 200 million disability-adjusted life-years,^1^ while the estimated number of deaths due to smokeless tobacco was over 650 000 in 2017.^3^ Ischemic heart disease (IHD), chronic obstructive pulmonary disease (COPD), cancer of trachea, bronchus, and lung, and stroke are responsible for the largest number of deaths attributable to smoking tobacco and account for approximately 72% of all deaths attributable to smoking tobacco^1^ Around 80% of the world’s 1.3 billion tobacco users live in low- and middle-income countries (LMICs).^2^ An estimated 77.5% of the total deaths attributable to smoking tobacco use occurred in LMICs in 2019.^1^ Finally, of the 71 countries in which the share of all-cause deaths that were due to smoking tobacco use increased significantly, 66 countries were LMICs.^1^ In addition, smokeless tobacco, which is commonly used in LMICs increases the risk of mortality due to all causes, all cancer, upper aero-digestive tract cancer, stomach cancer, cervical cancer, IHD and stroke, with 88% of the mortality burden being borne by the South-East Asian region.^3,4^ India accounted for 70%, Pakistan for 7% and Bangladesh for 5% DALYs lost due to smokeless tobacco use.^5^

Effective implementation of interventions for tobacco cessation can both increase healthy life expectancy and decrease health-care costs.^6^ There is substantial evidence demonstrating the effectiveness of a range of individual-level interventions for tobacco cessation, including brief advice, pharmacotherapy such as bupropion, varenicline and Nicotine Replacement Therapy (e.g., nicotine patch, nicotine gum), and a variety of psychological interventions.^7^ The latter include a wide variety of interventions such as brief counseling, individual or group cognitive-behavioral therapy, telephone counseling, combined pharmacotherapy and behavioral treatment, brief motivational interventions, and third-generation psychological treatments such as behavioral activation.^7,8^

This systematic review was situated in a larger intervention development program which aimed to design and test a contextually relevant behavioral intervention for tobacco cessation in India.^9^ Hence, while we acknowledge the role of pharmacological interventions in tobacco cessation, we focused on synthesizing the evidence on behavioral interventions to be able to extract data on effective intervention components that could inform the intervention that we were developing.

Studies from LMICs have shown the superiority of counseling compared to usual care/brief advice, the combination of bupropion and counseling compared to usual care, and brief advice compared to usual care in achieving abstinence.^10^ The paucity of studies assessing the efficacy of m-Health interventions for smoking cessation also creates a unique opportunity for further research in LMICs, as such interventions have the potential to significantly improve to overcome barriers to access.^11^

However, most of this evidence is from high-income countries (HICs) which is not easily transferable to LMICs, as patterns of tobacco use, health beliefs associated with tobacco use, and awareness of specific health risks vary substantially based on sociocultural context.^12^ While most evidence-based interventions focus on smoked tobacco, smokeless tobacco use is more common in LMICs, probably due to deep integration in sociocultural history, and may require different intervention approaches.^13^ Additionally, the proportion of tobacco users with intention to quit is considerably higher in HICs compared to LMICs.^14^ Finally, the feasibility of deploying evidence-based interventions from HICs for tobacco cessation might be limited in LMICs due to high costs.^15^ Hence, as indicated by existing evidence in other areas such as mental health and medication adherence for substance use disorders, culturally specific behavioral interventions may be cheaper and possibly more effective in achieving tobacco cessation in LMICs.^13,16^Although 80% of global tobacco-related deaths are reported in LMICs, only 1% of total global tobacco control expenditure is directed there.^17^ The majority of LMICs lack the numerous HICs’ decades-long expertise and ability to create, assess, and carry out tobacco control programmes.^18,19^ In many LMICs, health professionals have high rates of tobacco use, which is another barrier to spreading the right message to the public.,^20^ The growing evidence on the effectiveness of smoking cessation interventions across all LMICs is inconclusive because of the quality of studies.^13^ There have been some reviews of smoking cessation intervention effectiveness in LMICs but these had several limitations—all but one were focused on specific countries or regions, none included smokeless tobacco interventions across LMICs, which is more common than smoking in these countries, and none focused exclusively on behavioral interventions.^21–25^ A recent systematic review and network meta-analysis tried to identify the best nonpharmacological tobacco cessation interventions in Indian settings, however the analysis did not account for the adherence to these interventions postintervention delivery.^24^ Another systematic review and meta-analysis included studies which were performed in an ideal situation with a trained workforce and meticulous follow-up; thus stating inability to extrapolate the findings of these interventions to real-world situations.^25^ A particular emphasis on the effectiveness and execution of interventions in LMICs is necessary due to the persistent tobacco use in these areas and the particular difficulties in achieving tobacco cessation there.^18,19^ The analysis of the efficacy and effectiveness of the behavioral interventions in LMICs may provide more insight on the challenges and the strategies that can be implemented to tackle them, which may further guide the creation of a contextually appropriate but nonresource-intensive intervention to increase access to tobacco cessation interventions in low resource settings.^13,18^

Our systematic review addresses this gap by assessing the size, scope, and quality of evaluations of behavioral interventions for cessation of use of any type of tobacco in any LMIC. We also aimed to synthesize the effectiveness evidence using meta-analysis.

## Methods

### Design

Systematic review and meta-analysis. The review protocol was registered a priori on Prospero (registration ID CRD42020195679). We did not make any changes or deviations to the registered protocol.

### Eligibility Criteria

We included randomized controlled trials (RCTs), quasi-experimental trials and nonrandomized trials that evaluated behavioral interventions for tobacco (smoked or smokeless) cessation, stand-alone or in combination with pharmacological interventions, conducted in LMICs (as defined by the World Bank),^26^ and published in the English language, without any limits on publication year. Behavioral interventions were defined as interventions designed to achieve modifications to existing habits, activities, personal actions, or lifestyles. We excluded studies that did not have “abstinence” as one of the outcomes, exclusively focused on pharmacological interventions, delivered intervention in group settings or in the form of a seminar or included interventions for tobacco use comorbid with other substances or mental health disorders.

### Information Sources

We searched the following electronic databases: MEDLINE, EMBASE, PsycINFO, Global Health, Cumulative Index to Nursing and Allied Health Literature, LILACS (Latin American & Caribbean Health Sciences Literature), Cochrane Central Register of Controlled Trials (CENTRAL), and African Journal Online (AJOL). Our search strategy was based on combination of terms related to “tobacco” (e.g., tobacco, smoking), “behavioral intervention” (e.g., counseling, cessation), “LMICs” (e.g., developing country, specific names of LMICs) and “randomized controlled trial” (e.g., RCT, trial). A detailed search strategy is described in Supplementary Material 1. We inspected the reference lists of selected studies and relevant reviews for additional potential studies. We first conducted the search in July 2020 and repeated it in March 2023 to identify any new evidence.

### Selection Process

We imported all retrieved citations into a review management platform Covidence and removed the duplicates. Two researchers independently screened all identified abstracts (AN, LG) and full-text articles (LG, MS). Conflicts concerning study inclusion were resolved by a third reviewer.

### Data Extraction

The reviewers (LG, DB, PJ) then independently extracted data on participant characteristics, baseline tobacco use, intervention and comparator characteristics, tobacco use outcome, and physical and other qualitative or quantitative information on the intervention and delivery process. The two data extraction sheets were compared, and discrepancies were resolved through discussion.

### Risk of Bias and Quality Assessment

Risk of bias was assessed using the Cochrane Risk of Bias 2.0 tool for intervention studies for possible selection bias, performance bias, attrition bias, selective reporting bias, and other bias.^27^ Two reviewers independently assessed each included study for potential risk of bias and resolved the disagreements via discussion. The quality of the evidence was assessed using the GRADE approach^28^ to generate a “Summary of evidence” table for the primary outcome (abstinence). The quality of a body of evidence was rated based on the risk of bias, imprecision, inconsistency, indirectness, and other considerations (such as publication bias and large ES). We started rating the certainty of evidence in each domain as high and downgraded according to the assessments of these five domains. The rating was downgraded by one point in the presence of serious concerns and by two points in the presence of very serious concerns.^29^

### Outcome

The primary outcome of interest was abstinence at 6 months postintervention follow-up. We included studies that reported a dichotomous primary outcome and follow-up at or later than 6 months. When the studies reported both biochemically verified and self-reported abstinence, we included biochemically verified abstinence. For the multiple follow-up timepoints at or later than 6 months, we used data at 6 months follow-up.

### Analysis

The narrative synthesis included analysis of findings from the included studies, structured around variables such as the study settings, target population characteristics (including baseline tobacco use, quit attempts, willingness to quit), intervention outcome, intervention delivery process and contents of intervention, and moderators of effect within the trial. Where appropriate, meta-analysis was undertaken using STATA v17 to compare the effect between- (1) interventions versus usual care and (2) behavioral intervention of interest versus other interventions. Studies that measured point prevalence abstinence and continuous abstinence were analyzed separately. For the meta‐analyses, we chose to use random‐effects models because of the expected diversity in the interventions. We calculated an effect size (ES) and risk ratios (RR) with 95% confidence intervals (CIs) from the individual trials. When a study compared more than 2 active interventions with a control arm, we included the intervention with higher intensity in the meta-analysis. In all three studies that compared active interventions,^31,62,63^ the paper clearly indicated which of the interventions was of higher intensity. The studies where the follow-up period was not defined were excluded from the meta-analysis. As recommended by the Cochrane handbook as “highly desirable,”^29^ we performed a sensitivity analysis where studies with a high risk of bias were excluded from the meta-analysis to test the impact of studies with high risk of bias on the pooled intervention effect. We

We assessed for heterogeneity visually by inspecting the overlap of CIs on the forest plots and using the La’Abbe plot and quantified heterogeneity using the *I*² statistic. To indicate considerable statistical heterogeneity, we used a threshold of inconsistency of *I*^2^ ≥ 50%. We also assessed the studies for evidence of publication bias using funnel plots to investigate for evidence of small‐study effects for the overall comparisons.^29^

## Results

**Figure 1.**
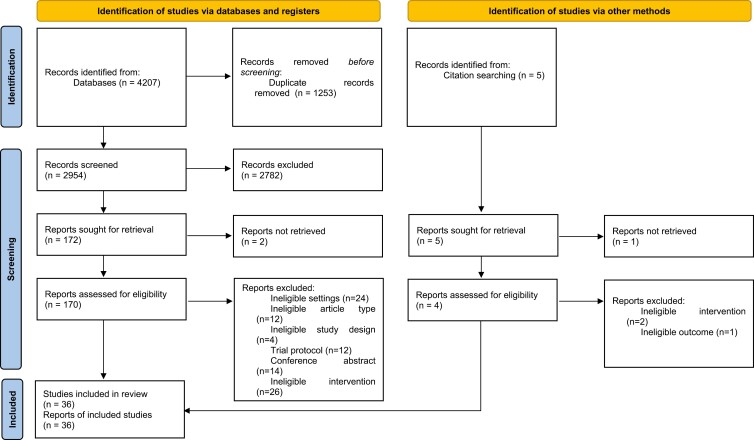
PRISMA 2020 flow diagram for study selection.

A total of 36 studies met the eligibility criteria for this review (Figure 1).

Supplementary Table S1 describes the characteristics of the 36 trials included in our review. Of these, 35 were RCTs, and one quasi-experimental study was embedded within a cluster RCT.^32^ Ten studies each were conducted in China^30,33–41^ and India,^42–51^ four in Turkey,^52–55^ three in Malaysia,^56–58^ two each in Brazil,^59,60^ and Pakistan^61,62^ and one each in Argentina/Uruguay,^31^ Iran,^63^ South Africa,^64^ Thailand,^65^ and Vietnam.^32^ A total of 32 283 adult and 424 adolescent participants were included in this review. Thirty-three of these studies aimed at smoking cessation and three studies targeted both smoked and smokeless tobacco. All, except one, of the studies were conducted with adult (≥18 years) participants. One study was conducted with school-going adolescents from grades 6 to 9.^46^ Most studies were conducted with adult tobacco users from the general population (*n* = 27), others with high-risk population groups such as people with tuberculosis,^42,45,61–64^ people with tuberculosis and HIV,^44^ and smokers with COPD.^36^ A few studies targeted their interventions towards parents or caregivers with the intention to reduce secondhand effects of tobacco use among children—pregnant women who smoked,^31^ parents or caregivers who smoked,^40,41^ and pregnant women whose husbands smoked.^35^ One study each directly targeted student tobacco users^46^ and prisoners who smoke.^49^ Four studies specifically targeted only males,^43,49,50,57^ and three targeted only females,^31,35,55^. In the remaining studies that did not restrict inclusion based on gender, a majority of participants (60% to 95%) were male. Participants were recruited from a diverse range of settings, including general public health clinics, specialized tobacco cessation centers, antenatal clinics, community clinics affiliated to teaching hospitals, university databases, online databases, and school and from the community through door-to-door screening.

The interventions delivered varied in terms of their mode of delivery, content, agent of delivery, frequency, and duration (Supplementary Table S1 and Supplementary Table S3). A majority of interventions were based on the World Health Organization’s (WHO) 5 As and 5 Rs tobacco cessation manual. Other manualized tobacco cessation treatments that were used were the ABC intervention (Ask, brief advice, and cessation support), guidelines by The Union’s (International Union against TB and Lung Disease) Smoking Cessation and Smoke-free Environments for TB Patients 2010, and the Contingency Management Competence Scale for Reinforcing Abstinence (CMCS). Other studies used theoretical frameworks or models such as the Transtheoretical Model, Motivational Interviewing or Enhancement, Theory of Planned Behavior, the Behavior Change Wheel, and Mindfulness Based Relapse Prevention to inform the interventions.

In terms of content, the interventions focused on educating tobacco users about the toxins in cigarettes and the negative effects on themselves and their families, encouraging goal setting related to tobacco cessation, setting quit dates and creating a detailed plan for the quit date, cognitive and behavioral strategies to control urges, identifying triggers, management of weight gain and strategies to prevent relapse. The mode of intervention delivery ranged from individual or group sessions in person, in-person sessions plus an educational printed handout, an initial in-person counseling session followed by text messages or phone calls reinforcing intervention messages, only text messaging or phone-based interventions. In-person sessions were delivered by a range of agents, including community health workers, Antenatal Care (ANC) providers, trained physicians, counselors, Directly Observed Treatment, Short-course (DOTs) facilitators, research assistants, and nurses. The duration of the intervention delivery ranged from a single 5-min session of brief advice to 18 months of in-person counseling.

The comparison groups typically received an intervention in the same mode as the intervention group but with different content or similar content of a lesser intensity. Six studies did not provide any intervention to the control group and three did not report any details of the comparison group intervention. Usual care was provided in five studies and included combination of nicotine gum and cognitive behavior therapy, or printed self-help materials, or unspecified usual care for tobacco cessation.

The primary outcome measure in 18 of the 36 studies was self-reported abstinence for a period ranging from 7 days to 12 months. The remaining 18 studies verified the self-reported abstinence rates with biochemical verification, using exhaled CO concentration, salivary cotinine or urinary cotinine concentration. Secondary outcomes measured were reduced exposure of children to CO, which was measured by using the urine cotinine test, number of quit attempts, daily consumption (to measure harm reduction), readiness to quit, and craving status (Supplementary Table S2).

We used the Cochrane risk of bias 2.0 tool to assess the quality of the studies included. Fourteen studies were found to have a high risk of bias, and sixteen had an unclear risk due to the unclear or problematic reporting of allocation and blinding of participants. Only six studies were found to have an overall low risk of bias.

### Meta-analysis

**Figure 2.**
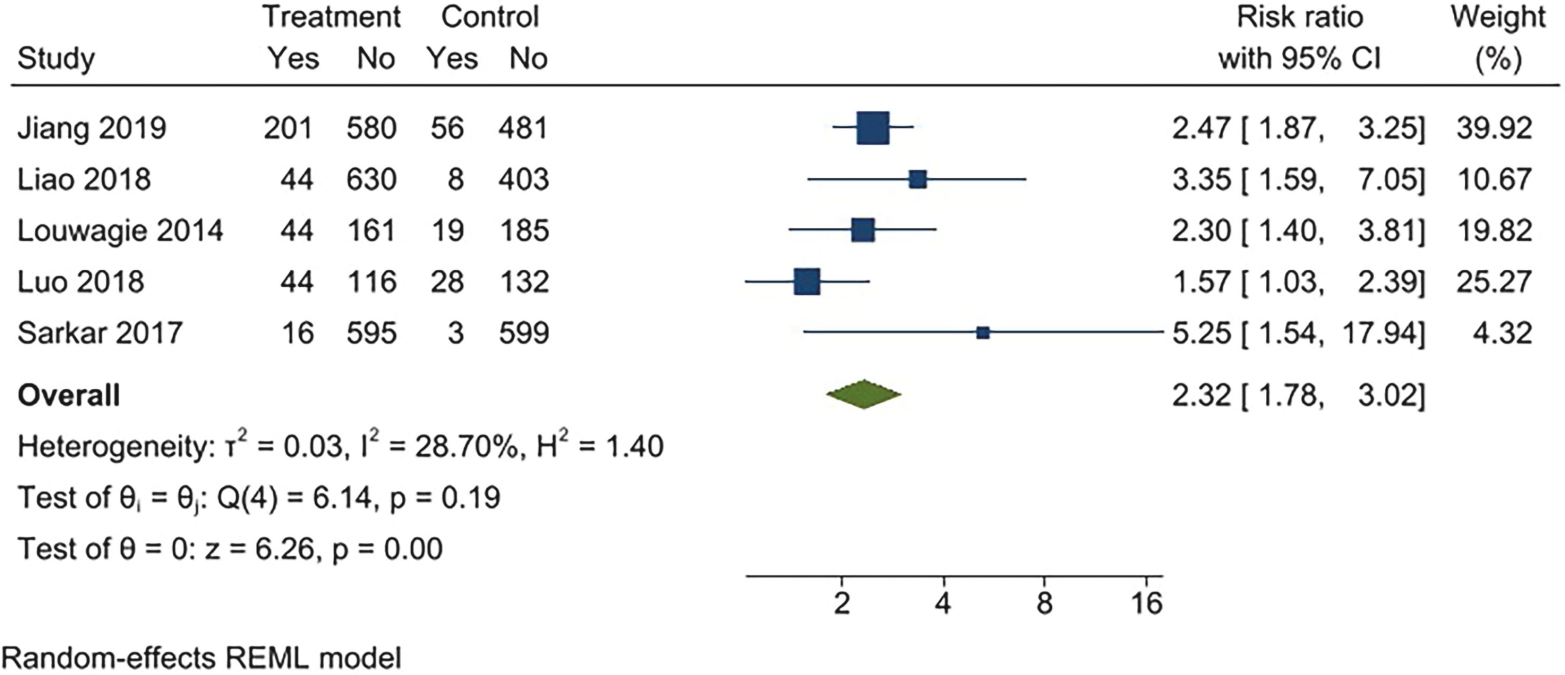
Behavioral intervention versus active intervention*. Outcome: 6-month continuous abstinence. *Active intervention included other behavioral intervention or pharmacological intervention.

**Figure 3.**
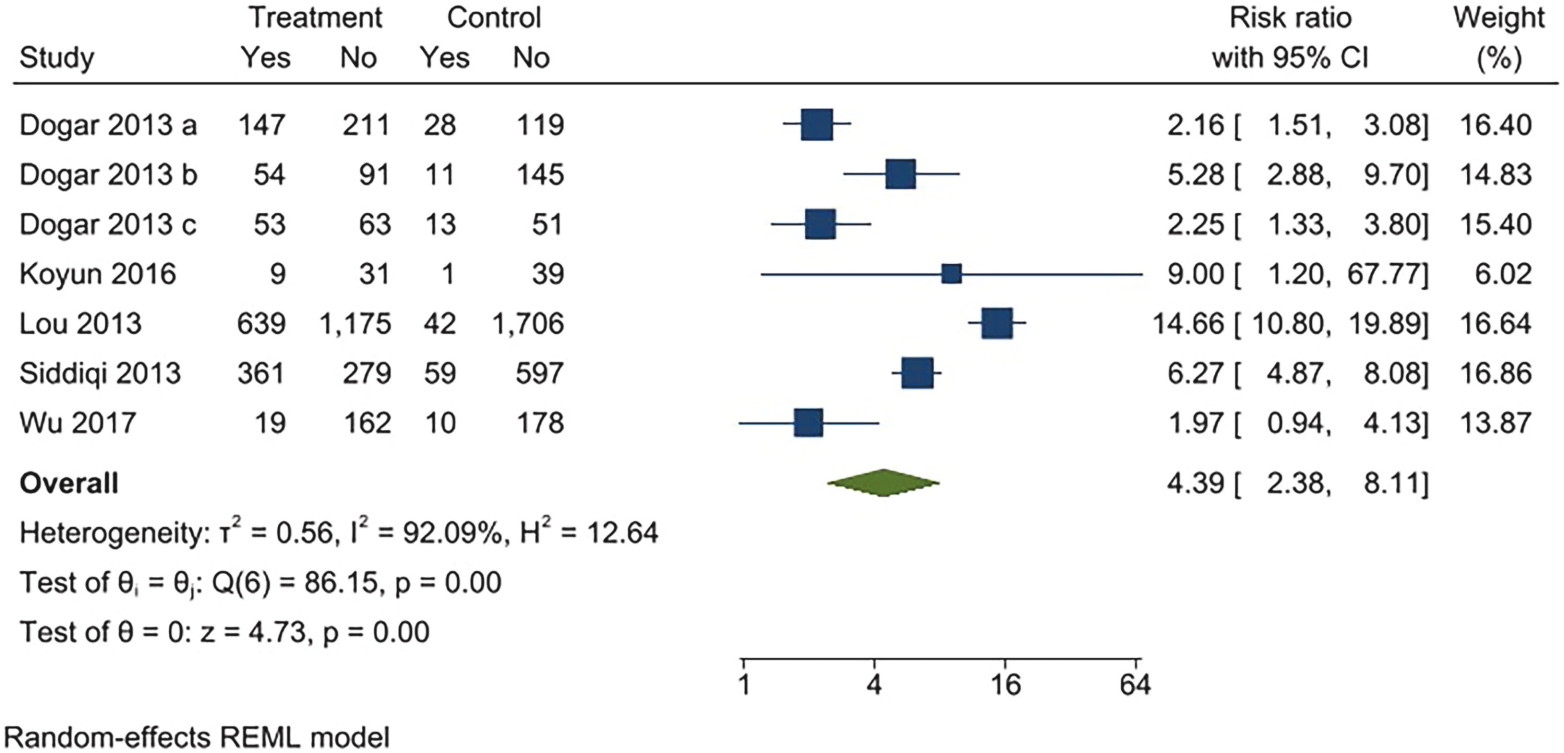
Behavioral intervention versus usual care*. Outcome: continuous abstinence. *Usual care included printed self-help materials or unspecified usual care for tobacco cessation. Dogar 2013a: participants smoking cigarettes exclusively; Dogar 2013b: participants smoking both cigarettes and hookas, Dogar 2013c: participants smoking hookah exclusively.

**Figure 4.**
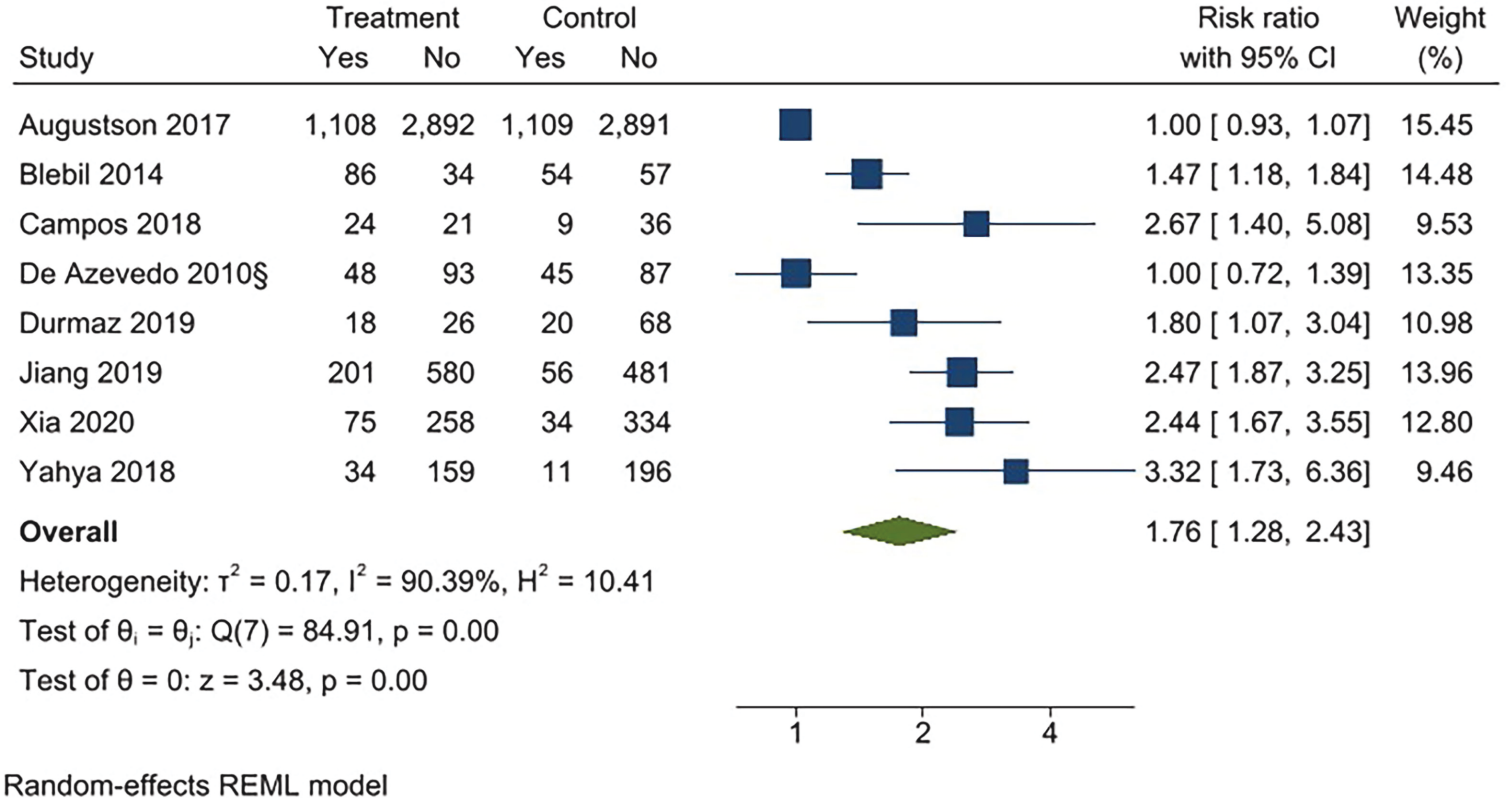
Behavioral intervention versus active intervention*. Outcome: Point prevalence abstinence at 6 months. *Active intervention included other behavioral intervention or pharmacological intervention.

**Figure 5.**
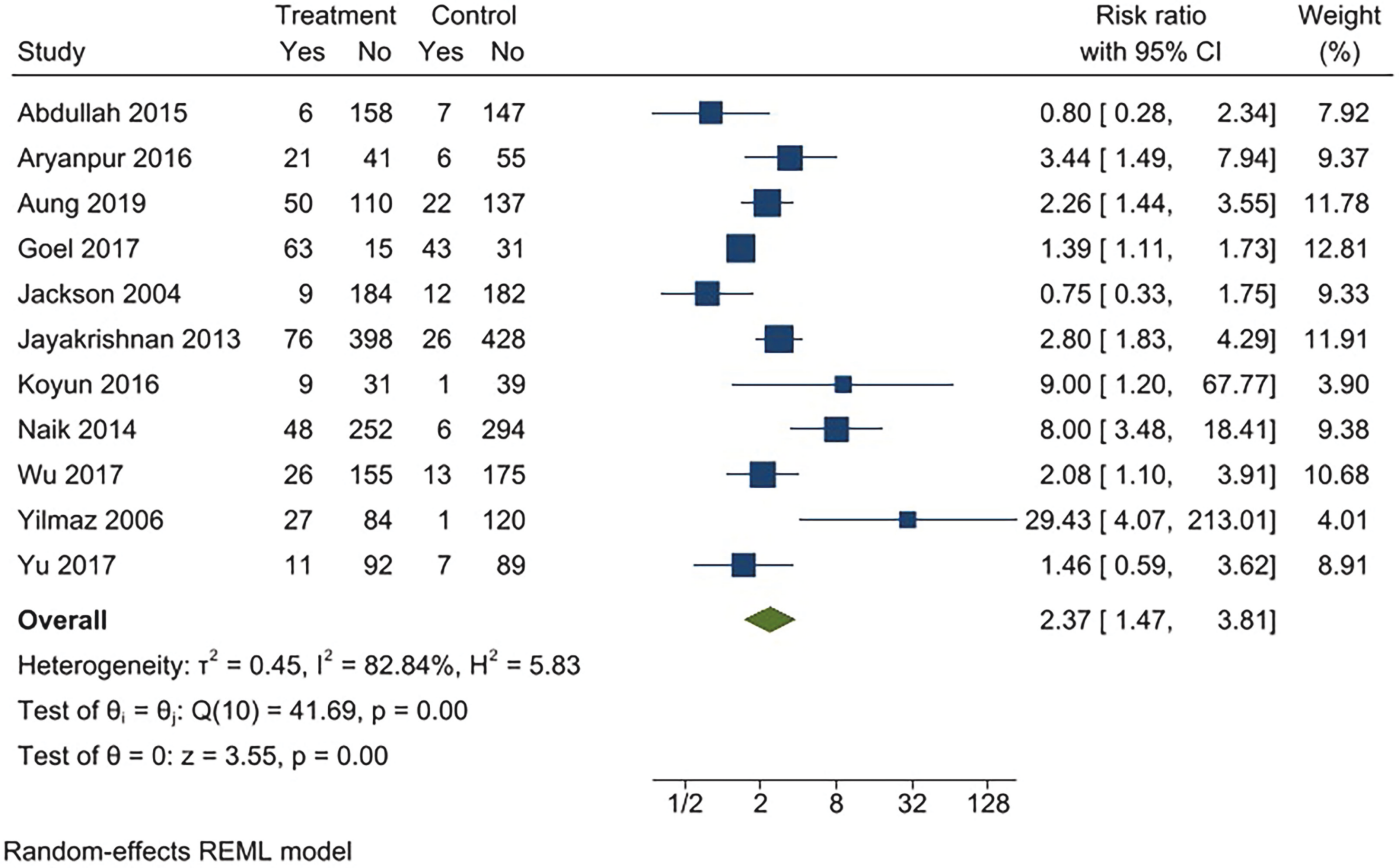
Behavioral intervention versus usual care*. Outcome: Point prevalence abstinence at 6 months. *Usual care included printed self-help materials or unspecified usual care for tobacco cessation.

**Figure 6.**
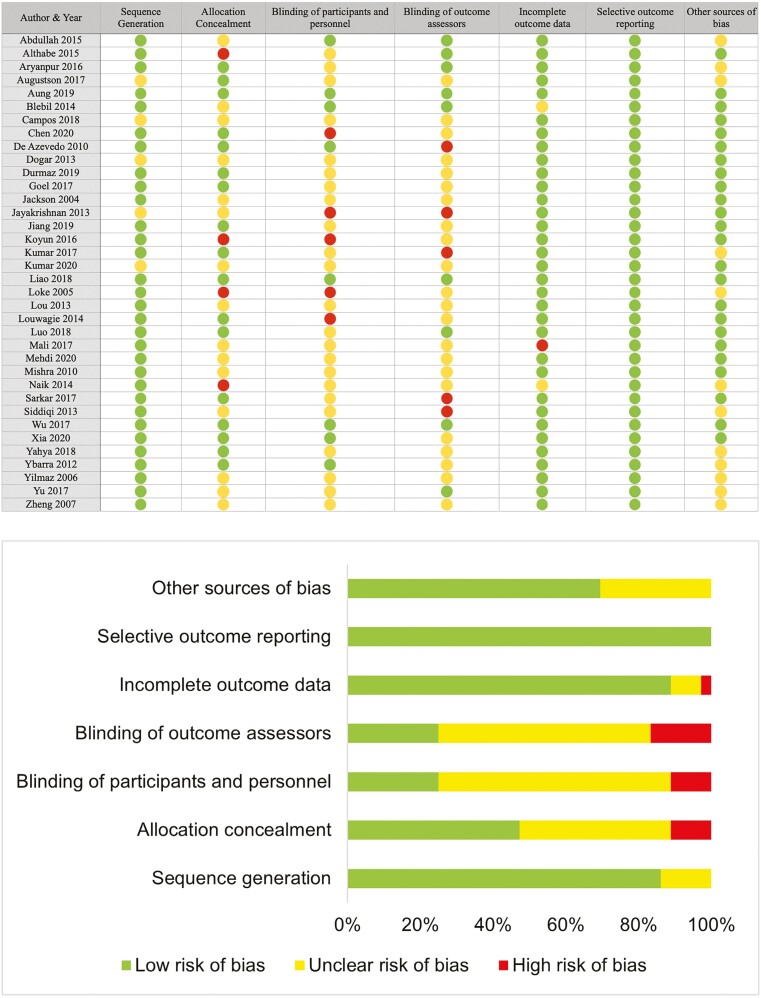
Quality of studies included in the review.

For continuous abstinence at 6 months, when pooled, the intervention arms were superior to both comparator arms that provided active interventions (RR 2.32; 95% CI 1.78 to 3.02), as well as usual care (RR 4.39; 95% CI 2.38 to 8.11) (Figures 2–5). Similar benefits were identified for point prevalence abstinence at 6 months, with intervention arms showing superiority over active intervention comparators (RR 1.76; 95% CI 1.28 to 2.44) and usual care (RR 2.37; 95% CI 1.47 to 3.81). The statistical heterogeneity was considerable for the comparison of continuous abstinence between the intervention arm and usual care (*I*^2^ = 92%) and point prevalence abstinence between the intervention arm and active interventions (*I*^2^ = 90%) and substantial for the comparison of point prevalence abstinence between intervention arm and usual care (*I*^2^ = 82%). For the comparison of behavioral interventions of interest and other interventions, the number of studies measuring 6-month continuous outcomes was too small to assess for asymmetry in the funnel plots for publication bias. For the other comparisons, the unequal scatter in the funnel plots indicates publication bias (Supplementary Figures S1–S4). Certainty of the evidence was high for continuous abstinence at 6 months compared to active interventions; very low for continuous abstinence at 6 months compared to usual care or no interventions and point prevalence abstinence at 6 months compared to active interventions; and moderate for point prevalence abstinence at 6 months compared to usual care or no interventions (Table 1; Figure 6).

**Table 1.** GRADE Summary of Evidence

Certainty assessment							№ of patients		Effect		Certainty	Importance
№ of studies	Study design	Risk of bias	Inconsistency	Indirectness	Imprecision	Other considerations	Behavioral interventions	other interventions	Relative (95% CI)	Absolute (95% CI)		
**Continuous outcome (compared to other intervention, follow-up: 6 months)**												
5	Randomized trials	Serious^a^	Not serious	Not serious	Not serious	Strong association	349/2431 (14.4%)	114/1914 (6.0%)	**RR 2.32** (1.78 to 3.02)	**79 more per 1000** (from 46 more to 120 more)	⨁⨁⨁⨁ High	CRITICAL
**Continuous abstinence (compared to usual care/no intervention, follow-up: 6 months)**												
5	Randomized trials	Very serious^b^	Serious^c^	Not serious	Not serious	Publication bias strongly suspected, strong association^d^	1282/3294 (38.9%)	164/2999 (5.5%)	**RR 4.39** (2.38 to 8.11)	**185 more per 1000** (from 75 more to 389 more)	⨁◯◯◯ Very low	CRITICAL
**Point prevalence abstinence (compared to other intervention, follow-up: 6 months)**												
8	Randomized trials	Serious^e^	Serious^f^	Not serious	Not serious	Publication bias strongly suspected^g^	1594/5657 (28.2%)	1338/5488 (24.4%)	**RR 1.76** (1.28 to 2.43)	**185 more per 1000** (from 68 more to 349 more)	⨁◯◯◯ Very low	CRITICAL
**Point prevalence abstinence (compared to usual care/no intervention, follow-up: 6 months)**												
11	Randomized trials	Serious^h^	Serious^i^	Not serious	Not serious	Strong association	319/1866 (16.7%)	144/1841 (8.4%)	**RR 2.37** (1.47 to 3.81)	**107 more per 1000** (from 37 more to 220 more)	⨁⨁⨁◯ Moderate	CRITICAL

CI = confidence interval; RR = risk ratio.

^a^Out of five publications, two were rated as having a high risk of bias, two as unclear risk of bias, and one as low risk of bias.

^b^Out of five, three publications are rated as having a high risk, one unclear risk of bias, and only one as low risk of bias.

^c^ *I* ^2^ value is 92.09%, *p* < .001. Effect sizes (EZs) vary among individual studies.

^d^Only one study on the right lower side of the funnel, and no study on the left lower side of the funnel.

^e^Out of eight publications, one was rated as having a high risk of bias, six unclear risk of bias, and one having a low risk of bias.

^f^ *I* ^2^ 90.39%, *p* < .001, EZs don’t vary considerably, and confidence intervals overlap in seven out of eight studies.

^g^The number of studies is not symmetrical on the two sides of the estimation line, and there are no studies on the lower left side.

^h^Out of 12, four publications are rated as having a high risk of bias, six having unclear risk of bias, and two having a low risk of bias.

^i^ *I* ^2^ value is 82.98, and *p* < .001.

### Sensitivity Analysis

Removing studies with a high risk of bias (*n* = 11) made no meaningful change in the size, direction, and significance of RR for the efficacy of behavioral interventions when compared with usual care or other interventions, apart from one comparison (intervention vs. usual care for continuous abstinence) which was no longer statistically significant, likely due to low power (Table 2).

**Table 2.** Effect Estimates According to Risk of Bias

Outcome	RR (95% CI), number of studies (n)	
	All studies	Studies with low and unclear risk of bias
Continuous abstinence (compared to usual care/ no intervention)	4.39 (2.38 to 8.11), *n* = 5	5.54 (0.78 to 39.50), *n* = 2
Continuous abstinence (compared to other intervention)	2.32 (1.78 to 3.02), *n* = 5	2.23 (1.53 to 3.24), *n* = 3
Point prevalence abstinence (compared to usual care/ no intervention)	2.37 (1.47 to 3.81), *n* = 11	1.86 (1.12 to 3.09), *n* = 7
Point prevalence abstinence (compared to other intervention)	1.76 (1.28 to 2.43), *n* = 8	1.92 (1.38 to 2.66), *n* = 7

## Discussion

Our review identified 36 studies evaluating the effectiveness of behavioral interventions for tobacco cessation in 13 LMICs. The diversity of reported outcomes limited the meta-analysis to 29 studies. The behavioral interventions of interest were superior to other active cessation interventions as well as usual care for both outcomes of interest—continuous abstinence and point prevalence abstinence at 6 months.

These are the first meta-analyses synthesizing the effectiveness of behavioral interventions for tobacco cessation in LMICs. What sets our review apart from these existing reviews is our focus on behavioral interventions delivered in LMICs. Our positive findings are consistent with other reviews which have synthesized evidence for tobacco cessation interventions. There is high‐quality evidence that individually delivered smoking cessation counseling can assist smokers to quit.^66^ Relevant to LMICs such as India, where smokeless tobacco is the more commonly used variant, varenicline, nicotine lozenges and behavioral interventions have been shown to be effective in helping smokeless tobacco users to quit.^67^ Focusing on specific types of professionals who might be in a more strategic position to deliver such interventions, there is evidence that tobacco abstinence rates increase in cigarette smokers if dental professionals offer behavioral support combined with pharmacotherapy.^68^ Similarly, when delivered in primary care, adjunctive counseling by an allied health professional, cost‐free smoking cessation medications, and tailored printed materials can increase the number of people who achieve smoking cessation.^69^ Within nonclinical settings, there is strong evidence that interventions directed towards individual smokers in the workplace increase the likelihood of quitting smoking, and these include individual and group counseling, as well as pharmacological treatment.^70^ Behavioral interventions delivered in-person or via telephone and used to supplement pharmacotherapy increase the chance of success of quitting by about 10% to 20%.^71^ Finally, with regard to co-occurring conditions, adding a psychosocial mood management component to a standard smoking cessation intervention increases long‐term cessation rates in smokers with depression.^72^

Despite the robustness of our methods, several issues need to be considered when making judgements about the applicability of these findings to programmes at scale. There were substantial variations in the interventions in terms of content, how they were delivered, how long they were delivered, and by whom. We observed wide variation in countries in terms of the reported number of studies. India and China reported a higher number of relevant tobacco cessation trials, while a single study was published from Indonesia- the second-largest cigarette retail market globally, and no relevant studies were reported from countries such as Bangladesh, which has a greater retail volume than India.^22^ This could limit generalizability of the findings for specific populations, intervention approaches etc. The pooled results were statistically and clinically heterogeneous, primarily due to the small number of studies and also the breadth of intervention characteristics; hence, these results need to be interpreted with caution.

Due to resource limitations, we excluded non-English language studies, which could have created bias. However, no relevant non-English studies were identified from the title or abstract screening, indicating that such a bias was unlikely to have affected the findings.

Most results from the included studies suggest interventions for tobacco cessation have a positive impact on patients’ smoking outcomes in LMICs, though the quality of evidence is limited. Given the variation in intervention content and delivery, there are still too few studies within each category to draw definite conclusions on specific intervention characteristics that influence effectiveness. Hence, while we identified a considerable number of relevant studies, a number of important research questions remain. Trialists need to describe interventions better to allow for better synthesis of evidence, conduct trials comparing interventions with different characteristics to better understand the effects of these variations, agree on standard measurement of outcomes to facilitate better pooling and comparison of data, and include economic data as that is an important consideration for health planning. Further systematic reviews need to draw on other study designs that examine process evaluations, economic evaluations and qualitative data to understand factors that are crucial for programme sustainability and equity of impact.

The Cochrane handbook for systematic reviews highlights the low power of tests for funnel plot asymmetry and is not recommended for meta-analyses where there are less than 10 studies.^29^ As only one of the meta-analyses in this review had more than 10 studies (*k* = 11), we deem using these tests as inappropriate. Our assessment is based on visual inspection of the forest plots and may not be a reliable measure of potential publication bias.

While behavioral interventions included in our analysis indicate effectiveness in reducing tobacco use in LMICs, more studies in LMICs are needed to arrive at definitive conclusions about generalizability.

## Supplementary Material

Supplementary material is available at *Nicotine and Tobacco Research* online.

ntae259_suppl_Supplementary_Appendix

## Data Availability

The data supporting the findings of this systematic review are derived from publicly available peer-reviewed publications. All relevant data are included in the cited studies within this review. No new primary data were generated or collected during the review process. The authors confirm that the data supporting the findings of this study are available within the article and/or its Supplementary Materials. For further details on the data used, please refer to the original sources provided in the reference list.
