# Supplementary material for: Behavioral Interventions for Tobacco Cessation in Low- and Middle-Income Countries: A Systematic Review and Meta-analysis
Source: Nicotine Tob Res. 2024 Nov 1;27(4):575–85. doi: 10.1093/ntr/ntae259 (PMC11931222; doi:10.1093/ntr/ntae259)
Supplement: ntae259_suppl_Supplementary_Appendix [file ntae259_suppl_supplementary_appendix.docx]

**Appendices**

## Supplementary Table 1: Details of trials included in the review

| **Author, Year** | **Country** | **Study design** | **Participants** | **Intervention (N)** | **Control (N)** | **Intervention** | **Control** |
| --- | --- | --- | --- | --- | --- | --- | --- |
| Abdullah, 2015 | China | RCT | Adult, Male (M), Female (F) | 164 | 154 | Brief advice | Child-development counselling and self-help materials |
| Althabe, 2015 | Argentina, Uruguay | Cluster RCT (CRT) | Adult F | 1562 | 1771 | Brief counselling based on 5As | Brief Seminar |
| Aryanpur, 2016* | Iran | RCT | TB patients, M, F | Brief advice- 62 | 61 | Brief advice plus Directly Observed Treatment, Short-course (DOTS) | Only Directly Observed Treatment, Short-course (DOTS) |
| Augustson, 2017 | China | RCT | Adult | 4000 | 4000 | High-Frequency Text Contact (HFTC)- m-health intervention | Low-Frequency Text Contact (LFTC) |
| Aung, 2019 | Thailand | RCT | Adult, M, F | 160 | 159 | Smoking cessation service package | Routine care for smoking cessation |
| Blebil, 2014 | Malaysia | RCT | Adult, M, F | 120 | 111 | Usual care (combination of nicotine  gum and cognitive behaviour therapy) plus phone counselling during 1^st^ month of quit attempt | Usual care (combination of nicotine  gum and cognitive behaviour therapy) |
| Campos, 2018 | Brazil | RCT | Adult, M, F | 45 | 45 | Intensive cognitive behaviour therapy (oral intervention 10 minutes plus educational video 30 minutes) | Brief oral intervention for 10 minutes |
| Chen, 2020 | China | RCT | Adult, M | 40 | 40 | Full version of the SCAMPI program (behavioural intervention program delivered through WeChat based text message) | Restricted version of the SCAMPI program (contact information for standard smoking cessation care, e.g. Quitline  in China, local smoking cessation clinics) |
| De Azevedo, 2010 | Brazil | RCT | Adult, M, F | 141 | 132 | High-intensity intervention (HII): Individual counselling ((motivational interview, plus behavioural  self-management techniques) for 30 minutes | Low-intensity intervention (LII)^#^: Individual counselling (advise to stop smoking) for 15 minutes |
| Dogar, 2013^‡^ | Pakistan | CRT | TB patients, M, F | 640 | 656 | Brief behavioural support sessions (BSS): two structured consultations  based on the World Health Organization’s “5 As Approach” plus self-help leaflet | Usual care (self-help leaflet) |
| Durmaz, 2019 | Turkey | RCT | Adult, M, F | 44 | 88 | Face-to-face individual counselling at first contact for 45 minutes, motivational interview or quitting counselling and WhatsApp text messages, plus support booklet | Usual care (face-to-face individual counselling at first contact 45 minutes, motivational interview or quitting counselling, depending on their intention to quit, plus support booklet) |
| Goel, 2017 | India | CRT | TB patients, M, F | 78 | 74 | ABC intervention: Ask, brief advice, and cessation support for 2–5 min | No intervention |
| Jackson, 2004 | Malaysia | RCT | Adult, M | 193 | 194 | Four questions on knowledge and beliefs about smoking, standardized verbal advice against smoking, and information leaflet | No intervention |
| Jayakrishnan, 2013 | India | RCT | Adult, M | 474 | 454 | Educational material, group & individual counselling | Educational material |
| Jiang, 2019 | Vietnam | Quasi-experimental study embedded in CRT | Adult, M, F | 781 | 537 | Ask, advise to quit, Assess readiness to quit, and Assist (brief counselling and educational materials) (4As) plus referral for in-person counselling sessions | 4As |
| Koyun, 2016 | Turkey | RCT | Adult, F | 40 | 40 | Trans-Theoretical Model (TTM)-based training and individual counselling for 45–60 minutes and self-help material | No intervention^¤^ |
| Kumar, 2017 | India | RCT | Adult, M, TB & HIV patients | 80 | 80 | Physician’s advice using modified 5As approach, brochure with smoking cessation information, and counselling | Brochure with smoking cessation information and counselling |
| Kumar, 2020^Ψ^ | India | CRT | TB patients, M | 170 | 195 | Enhanced motivation counselling (Brochures on Smoking Cessation provision, flip charts, posters, movie/video presentations, family counselling) | Standard motivation (5 A approach and 5R approach) |
| Liao, 2018 | China | RCT | Adult, M, F | High Frequency Messaging (HFM): 674  Low Frequency Messaging (LFM): 284 | 411 | HFM: Happy Quit program motivational messages and behaviour change techniques, information relevant to quitting (3-5 messages per day for 12 weeks, followed by 3 to 5 per week for 12 weeks)  LFM: Happy Quit program booklet after completion of the trial (3-5 messages per week for 12 weeks, followed by to 1- 2 per week for 12 weeks) | Text messages unrelated to quitting (1 text message per week) |
| Loke, 2005 | China | RCT | Pregnant women | 380 | 378 | Standardised advice, educational booklet, health reminders | No intervention |
| Lou, 2013 | China | RCT | COPD patients, M | 1,814 | 1,748 | Brief advice with plan to quit smoking | Usual care |
| Louwagie, 2014 | South Africa | RCT | TB patients, M, F | 205 | 204 | Brief MI, and Smoking cessation booklet | Short standardized smoking cessation message, and smoking cessation booklet |
| Luo, 2018 | China | RCT | Adult, M, F | 160 | 160 | 5As+5Rs counselling during hospitalization, telephone counselling and self-help materials | Motivational interventions based on PHS 5Rs |
| Mali, 2017 | India | CRT | Students, M, F | 402 | 422 | Peer delivered ASSIST intervention | No intervention |
| Mehdi, 2020 | India | RCT | Adult, M, F | 37 | 38 | Combined Pharmacotherapy + brief (45 minutes) individual psychotherapy  session for tobacco cessation based on 5A | Pharmacotherapy |
| Mishra, 2010 | India | RCT | Adults | Arm1: 111 Arm 2: 188 Arm 3: 194 |  | Arm 2: Active Health Education (HE) sessions followed by focus group discussion (FGD)  Arm 3 : Active HE sessions followed by FGD and Behavioural Therapy (BT) in the form of one-to-one counselling | Arm 1: Health education pamphlets |
| Naik, 2014 | India | RCT | Adult, M | 300 | 300 | Motivational interviewing | Not reported |
| Rajanandh, 2012 | India | RCT | Adult, M | 40 | 40 | Motivational enhancement counselling | Usual care |
| Sarkar, 2017 | India | CRT | Adult, M, F | 611 | 602 | Single session tobacco quit advice (15 minutes), and yogic breathing exercises. | Very brief quit advice (1 minute) |
| Siddiqi, 2013^∑^ | Pakistan | CRT | TB patients, M, F | BSS: 640 | 656 | Behavioural support session (BSS): World  Health Organization’s “5 A’s” approach (11) and included behaviour change technique, 2 structured sessions using an educational flipbook | Usual care and self-help leaflet |
| Wu, 2017 | China | RCT | Adult | 181 | 188 | Smoking-reduction intervention: one face-to-face advice plus 5 telephone follow-up interventions; Very brief advice, followed by additional advice at follow up visits | Exercise- and diet-advice: one face-to-face advice plus 5 telephone follow-up interventions |
| Xia, 2020 | China | RCT | Adult, M | Video: 333. Text: 322 | 368 | Video group: brief advice, 4 videos on various risks of smoking, content developed using the theory of planned behaviour.  Text group: brief advice,4 text messages on various risks of smoking, content developed using the theory of planned behaviour | Brief advice on smoking cessation and a leaflet |
| Yahya, 2018 | Malaysia | RCT | Adult, M, F | 193 | 207 | 5A’s & 5R’s strategies | Brief advice |
| Ybarra, 2012 | Turkey | RCT | Adult, M, F | 76 | 75 | SMS Turkey (text messaged based on CBT) | General quitting information brochure |
| Yilmaz, 2006 | Turkey | RCT | Mothers of a child <  16 years, F | Child intervention- 111 Mother intervention- 131 | 121 | Child intervention: risks to children’s health were explained. mother intervention: risks of tobacco to the mother’s own health was discussed. | General personal health information |
| Yu, 2017 | China | RCT | Adult, M, F | Fathers:  Group A- 103 Group B- 100 | Fathers: 96 | Group A: in-person counselling, education on establishing a smoke-free home, table tents and posters.  Group B: Educational intervention, Text message intervention | No intervention |

CRT= Cluster randomized trial; RCT= Randomized controlled trial

*The combined group received pharmacotherapy for tobacco cessation along with DOTS and behavioural intervention. Hence, it was not included in this systematic review and meta-analysis.

^#^ The usual care was provided to participants who were lost to randomization. Hence, we have not reported data for this arm.

^‡^The BSS+ group received pharmacotherapy for tobacco cessation along with the behavioral intervention. Hence, it was not included in this systematic review and meta-analysis.

^¤^TTM-based self-help material was provided after end of last follow up interview.

^Ψ^The intervention arm containing pharmacotherapy was not included in this systematic review and meta-analysis.

^∑^BSS+ group was not included in this review, as it also included pharmacotherapy.

## **Supplementary Table 2: Outcome measures and effects of intervention in individual studies**

| Author, Year | Outcome | Outcome Measures | Reported As | Timepoint | Intervention | Control | p |
| --- | --- | --- | --- | --- | --- | --- | --- |
| Abdullah, 2015 | Smoking cessation | Biochemical verification of self-reported smoking cessation | n (%) | 6 months follow up | 6 (6.1) | 7(8.5) | 0.533 |
| Althabe 2015 | Quit smoking during pregnancy* | Biochemical verification of self-reported smoking cessation | n (%) | Follow up period not reported | 52 (10.9) | 33 (8.1) | 0.239 |
| Aryanpur 2016 | Smoking cessation | Biochemical verification (Expiratory CO concentration <7ppm: non-smokers; >7ppm: smokers) | n (%) | 6 months follow up | BA- 21 (33.9) | 6 (9.8) | <0.001 |
| Augustson 2017 | 7-day point prevalence abstinence (PPA)^ | Self-reported | n (%) | Immediately after intervention | 1114 (27.9) | 1069 (26.7) | 0.259 |
|  |  |  |  | 1 month follow up | 1218 (30.5) | 1216 (30.4) | 0.961 |
|  |  |  |  | 3 months follow up | 1067 (26.7) | 1123 (28.1) | 0.160 |
|  |  |  |  | 6 months follow up | 1108 (27.7) | 1109 (27.7) | 0.980 |
| Aung 2019 | Smoking cessation | Biochemical verification of self-reported smoking cessation | n (%) | 6 months follow up | 50 (31.3) | 22 (13.8) | <0.001 |
|  |  |  |  | 1 year follow up | 62 (38.8) | 23 (14.5) | < 0.001 |
|  |  |  |  | Cessation for 6 months, at 1 year follow up^#^ | 41 (25.6) | 18 (11.3) | < 0.001 |
| Blebil 2014 | 4-week PPA | Biochemical verification of self-reported abstinence | n (%) | 3 months follow up | 56 (46.7) | 41 (36.9) | 0.144 |
|  |  |  |  |  |  |  |  |
|  |  |  |  | 6 months follow up | 86 (71.7) | 54 (48.6%) | <0.001 |
| Campos 2018 | Abstinence | Biochemical verification of self-reported abstinence | n (%) | 6 months follow up | 24 (53.3) | 9 (20) | 0.001 |
| Chen 2020 | 30-day smoking abstinence | Biochemical verification of self-reported abstinence | n (%) | 6 weeks follow up | 10 (25%) | 2 (5%) | 0.03 |
| De Azevedo 2010^§^ | 7-day PPA | Self-reported | n (%) | 6 months follow up | 48 (44.9) | 45 (41.7) | 0.03^‡^ |
| Dogar 2013^§^ | Continuous smoking abstinence | Biochemical verification  (Expired CO ≤9 ppm: abstinence) | n (%) | 6 months follow up | Cigarette (data analysed for N=358): 147 (41.1)) | Cigarette (data analysed for N=395): 28 (7.1) | 0.02^∆^ |
|  |  |  |  |  | Mixed (cigarette+ hookah; data analysed for N=146): 54 (37.0) | Mixed (cigarette + hookah; data analysed for N=156): 11 (7.1) |  |
|  |  |  |  |  | Hookah (data analysed for N=116): 53 (45.7) | Hookah (data analysed for N=64): 13 (20.3) |  |
|  |  |  |  |  |  |  |  |
| Durmaz 2019 | Abstinence | Self-reported | n (%) | 1 month follow up (PPA) | 29 (65.9) | 36 (40.9) | <0.05^∞^ |
|  |  |  |  | 3 months follow up (continuous) | 22 (50) | 27 (30.7) | <0.05^∞^ |
|  |  |  |  | 6 months follow up (continuous) | 18 (40.9) | 20 (22.7) | <0.05^∞^ |
| Goel 2017 | 2-week PPA | Self-reported | n (%) | 6 months follow up | 63 (80.2) | 43 (57.5) | NR |
| Jackson 2004 | Quit rate | Biochemical verification of self-reported quitting | n (%) | 6 months follow up | 9 (4.7) | 12 (6.2) | NR |
| Jayakrishnan 2013 | PPA | Self-reported | n (%) | 6 months follow up | 76 (16) | 26 (5.7) | 0.0001 |
|  |  |  |  | 12-months follow up | 70 (14.7) | 31 (6.8) | NR |
| Jiang 2019 | Smoking abstinence rate | Biochemical verification | n (%) | 6 months follow up | 201 (25.7) | 56 (10.5) | < 0.001 |
| Koyun 2016 | Smoking cessation rate | Self-reported | n (%) | 6 months follow up | 9 (23.7) | 1 (2.6) | <0.05 |
| Kumar 2017 | Smoking cessation | Biochemical verification of self-reported cessation | n (%) | 1 month follow up | 28 (35) | 24 (30) | 0.480 |
| Kumar 2020 | Smoking quit rate | Self-reported | n (%) | 2 months follow up^§^ | 99 (Data analysed for N=149, 66.4) | 80 (Data analysed for N=169, 47.3) | <0.001 |
|  |  |  |  | 6 months follow up^§^ | 96 (Data analysed for N=116, 82.8) | 75 (Data analysed for N=145, 51.7) | <0.001 |
| Liao 2018 | Continuous smoking abstinence | Biochemical verification of self-reported cessation | n (%) | 24 weeks (end of intervention) | HFM: 44 (6.5) | 8 (1.9) | <0.001 |
|  |  |  |  |  | LFM: 17 (6) |  | 0.002 |
|  |  | Self-reported | n (%) | 24 weeks (end of intervention) | HFM: 46 (6.8) | 8 (1.9) | <0.001 |
|  |  |  |  |  | LFM: 18 (6.3) |  | 0.004 |
|  | 7-day PPA | Self-reported | n (%) | 24 weeks (end of intervention) | HFM: 130 (19.3) | 27 (6.6) | <0.001 |
|  |  |  |  |  | LFM: 55 (19.4) |  | <0.001 |
| Loke 2005 | Abstinence from cigarettes | Self-reported | n (%) | Past 7 days at 3-5 months follow up | 32 (8.4) | 18 (4.8) | 0.04 |
|  |  |  |  | Past ≥1 month at 3-5 months follow up | 23 (6.1) | 16 (4.2) | 0.26 |
| Lou 2013 | Continuous abstinence from smoking ^£^ | Biochemical verification of self-reported cessation^§^ | n (%) | From the start of month 24 to the end of month 30 (6 months follow up) | 639 (46.4) | 42 (3.4) | <0.001 |
|  |  |  |  | 12 months follow up | 630 (45.8) | 49 (4) | <0.001 |
|  |  |  |  | 18 months follow up | 622 (45.2) | 57 (4.6) | <0.001 |
|  |  |  |  | 24 months follow up | 610 (44.3) | 63 (5.1) | <0.001 |
| Louwagie 2014 | Sustained abstinence | Self-reported | n (%) | 6-month | 44 (21.5) | 19 (9.3) | NR |
| Luo 2018 | Continuous abstinence | Biochemical verification (Exhaled carbon monoxide levels ≤10 ppm) | n (%) | Weeks 9–12 | 44 (27.5) | 28 (17.5) | 0.032 |
| Mali 2017 | Cessation of smokeless tobacco use^$^ | Self-reported | n (%) | 6 months follow up | 60 (14.9) | 46 (10.9) | NR |
|  |  |  |  | 12 months follow up | 88 (21.9%) | 57 (13.5) | NR |
| Mehdi 2020 | Continuous abstinence | Self-reported | n (%) | 6 weeks follow up | 18 (47.3) | 10 (26.3) |  |
|  |  |  |  |  |  |  | NR |
|  |  |  |  | 8 weeks follow up | 20 (54.1) | 11 (28.9) |  |
|  |  |  |  | 12 weeks follow up | 20 (54.1) | 11 (28.9) |  |
| Mishra 2010 | Quit tobacco | Self-reported | n (%) | Follow up period not reported | Arm 2: 37 (20) Arm 3: 38 (19) | Arm 1: 7 (6) | NR |
| Naik 2014 | Smoking cessation | Self-reported | n (%) | 6 months follow up | 48 (16) | 6 (2) | NR |
| Sarkar 2017 | 6 months sustained abstinence | Biochemical verification of self-reported cessation | n (%) | 7 months follow up | 16 (2.6) | 3 (0.5) | 0.013 |
| Siddiqi 2013^∑^ | Continuous smoking abstinence | Biochemical verification | n (%) | 6 months follow up^§^ | BSS: 361 (58) | 59 (9.4) | <0.001 |
| Wu 2017 | 6-month prolonged abstinence at 12 months | Self-reported | n (%) | 12 months follow up | 19 (15.7) | 10 (7.8) | 0.062 |
|  | Abstinence | Biochemical verification | n (%) | 12 months follow up | 11 (6.1) | 4 (2.1) | 0.07 |
|  | 7-day point prevalence | Self-reported | n (%) | 6 months follow up | 26 (14.4) | 13 (6.9) | 0.02 |
|  | 7-day point prevalence | Self-reported | n (%) | 12 months follow up | 24 (13.3) | 13 (6.9) | 0.049 |
|  | 1 month prolonged abstinence | Self-reported | n (%) | 6 months follow up | 25 (13.8) | 11 (5.9) | 0.01 |
|  | 3-month prolonged abstinence | Self-reported | n (%) | 6 months follow up | 22 (12.2) | 12 (6.4) | 0.053 |
| Xia 2020 | 7-day PPA | Expired CO <4 ppm | n (%) | 6 months follow up | Video 75 (22.5) | 34 (9.2) | *<*0.001 |
|  |  |  |  |  | Text 48 (14.9) |  | 0.02 |
|  |  | Self-reported^Ω^ | n (%) |  | Video 82 (24.6) | 42 (11.4) | 0.02 |
|  |  |  |  |  | Text 56 (17.4) |  |  |
| Yahya 2018 | Smoking abstinence | Self-reported | n (%) | 1 month follow up | 32 (16.6) | 16 (7.7) | 0.006 |
|  |  |  |  | 3 months follow up | 36 (18.7) | 21 (10.1) | 0.015 |
|  |  |  |  | 6 months follow up | 34 (17.6) | 11 (5.3) | <0.001 |
| Ybarra 2012 | Sustained abstinence | Biochemical verification of self-reported abstinence (CO ≤8ppm) | n (%) | 3 months follow up | 11 (8) | 5 (4) | NR |
|  |  | Biochemical verification of self-reported abstinence (CO ≤8ppm) | n (%) | 7 days at 4 weeks | 12 (9) | 9 (7) | NR |
|  | PPA |  |  |  |  |  |  |
|  |  | Self-reported | n (%) | 7 days at 3 months | 13 (10) | 5 (4) | NR |
|  |  |  |  | 30 days at 3 months | 11 (8) | 5 (4) | NR |
| Yilmaz 2006 | Quit smoking | Self-reported | n (%) | 6 months follow up | Child intervention 27 (24.3) | 1 (0.8) | 0.0001^Ɵ^ |
|  |  |  |  |  | Mother intervention 17 (13) |  |  |
| Yu 2017 | Smoking cessation in fathers | Self-reported | n (%) | 6 months follow up | Group A: 11 (10.7) Group B: 20 (20.0) | 7 (7.3) | Group A vs control: 0.408  Group B vs. control: 0.012 |
|  |  |  |  | 12 months follow up | Group A: 17 (16.7) Group B: 22 (22.7) | 9 (9.7) | Group A vs control: 0.156  Group B vs. control: 0.018 |

CO= Carbon monoxide; PPA= Point prevalence abstinence

*The outcome “Quit smoking during pregnancy” was calculated considering as denominators the women who quit smoking during pregnancy plus the women who continued smoking during pregnancy.

^The main analyses reported in this study are based on intent-to-treat principle, hence only those are included in this review and meta-analysis.

^#^This was measured via the smoker’s self-reporting of smoking cessation over the previous 24 h, self-reporting of smoking cessation over the previous seven days, and a confirmatory measurement of carbon monoxide in parts per million (ppmCO) using a piCo+ Smokerlyzer. These measurements were longitudinally conducted at baseline, 3 month follow up and 6month follow up to confirm behavior change. At each follow up, the participants undergo all the outcome measurement. When someone cannot stop continuously from zero to sixth months of follow up, these cases were not considered as smoking cessation.

^§^Per-protocol analysis.

^£^Continuous abstinence as a participant report of zero cigarettes per day for at least 6 months and confirmed by exhaled carbon monoxide values of 10 parts per million.

^‡^p-value was reported to compare outcome between 3 arms: high-intensity intervention, low-intensity intervention and usual care.

^∆^p-value was reported to compare the outcome between 2 arms and all 3 groups: cigarette, mixed (cigarette + hookah) and hookah.

^∞^Reported from the multivariate logistic regression analysis, with control group as reference.

^Ɵ^p-value was reported to compare outcome between 3 arms: child intervention, mother intervention and control group.

^Ω^Results at timepoints less than 6 months were not extracted in this systematic review and meta-analysis.

^∑^BSS+ group was not included in this review, as it also included pharmacotherapy.

^$^Values were calculated by subtracting the number of smokeless tobacco users at follow up from the number of smokeless tobacco users at baseline. The denominators for calculating percentages were the number of participants at baseline.

## Supplementary Table 3: Details of intervention and control conditions

| **Author,**  **Year** | **Intervention** | | | | **Comparison** | | | | **Follow- up** |
| --- | --- | --- | --- | --- | --- | --- | --- | --- | --- |
|  | **Type** | **Delivery Mode** | **Delivery Agent** | **Duration** | **Type** | **Delivery Mode** | **Delivery Agent** | **Duration** |  |
| Abdullah, 2015 | Brief advice to quit smoking, The counselling approach was patient centered, which means that the counsellor engaged the households in a discussion about smoking and SHS exposure to the child in a non-threatening manner and engaged the household members in making decisions about the counsellor’s recommendations. | in-person and telephonic counselling, with printed self-help materials | Community health workers (CHWs) who received a 3-day practicum training. | 4 months | Placebo intervention | in-person and telephonic counselling, with printed self-help materials | Community health workers (CHWs) | 4 months | 6 months after initial contact |
| Althabe 2015 | Brief smoking cessation counselling based on 5As | In-person | ANC providers | 14-18 months | Brief Seminar | In-person | ANC providers | Not reported | "The last 6 months of the 18-month intervention (follow up) |
| Aryanpur, 2016 | BA group- The educational intervention based on the manual of smoking cessation interventions for TB patients, additional to the DOTS course  Combined intervention group- 1. The educational intervention based on the manual of smoking cessation interventions for TB patients, additional to the DOTS course 2. slow-release bupropion | In-Person | one trained physician in each center | "Counselling- 2 weeks  Bupropion- 9 weeks" | only DOTS regimen | In-person | Not reported | Not reported | end of the second, third and fourth counselling sessions, end of the second, fourth and sixth months |
| Augustson 2017 | HIGH-FREQUENCY TEXT CONTACT (HFTC)- m-health intervention | Text messages | Not reported | 1 week of pre–quit day messages to all participants. and 6 weeks after this week. | LOW-FREQUENCY TEXT CONTACT (LFTC) | Text messages | Not reported | 6 weeks | 1,3, 6 months |
| Aung 2019 | smoking cessation service package | in-person | hospital nurse | 3 months | existing routine service for smoking cessation | In-person | hospital healthcare worker | first visit | 6 months, 12 months |
| Blebil 2014 | usual care procedure plus extra counselling sessions through phone | in-person, printed materials | Counsellor | 3 months | usual care recommended by the Ministry of Health, Malaysia | In-person | Counsellor | 3 months | 3 months and 6 months from last visit |
| Campos 2018 | Brief intervention | in-person | Counsellor | 10 minutes | Intensive cognitive behavioral therapy-based intervention | in-person, video presentation | Counsellor | 40 min | 1, 3, and 6 months after discharge from the hospital |
| Chen 2020 | The SCAMPI program was designed using the behaviour change wheel (BCW) framework, a theory- and evidence-based tool for designing interventions based on an analysis of the nature of the behaviour, the mechanisms that need to be changed to bring about behaviour change, and the interventions and policies required to change those mechanisms. Participants randomised to the intervention group had access for 6 weeks to the full version of the SCAMPI program  WeChat based Text message intervention | Text messages | - | 42 | participants randomized to the control group had access for 6 weeks to a restricted version of the SCAMPI program. | Text messages | - | 42 | At 4 weeks and 6 weeks |
| deAzevedo 2010 | low-intensity intervention (LII)- individual counselling session for 15 minutes   high-intensity intervention (HII)- individual counselling session for 30 minutes | Intervention: LII- in-person  HII- in-person, telephonic | trained smoking-cessation counsellor | LII- 15 minutes  HII- 30 minutes in-person, telephonic- 10 minutes | usual care (UC) | In-person | hospital staff | Not reported | 6 months |
| Dogar 2013 | BSS: structured consultations based on the World Health Organization’s “5 As Approach”, and using BCTs considered effective in smoking cessation   BSS+: structured consultations based on the World Health Organization’s “5 As Approach”, plus sustained-release bupropion | In-Person | TB DOTS (directly observed treatment, short-course) paramedics- nurses or auxiliary nurses | BSS- 2 sessions,  - 30 minutes &  10 minutes  BSS+- BSS, and Bupripion 7 weeks | usual care (UC) | Not reported | Not reported | Not reported | 5- and 25-week post- intervention follow-up visits |
| Durmaz 2019 | WhatsApp messages plus usual care | in-person, telephonic + WhatsApp messages | physicians trained on quitting tobacco | 3 months | usual care (UC) | in-person, telephonic, if needed | physicians trained on quitting tobacco | 3 months | 1st, 3rd and 6th month |
| Goel 2017 | ABC intervention: Ask, brief advice, and cessation support: the International Union against TB and Lung Disease (The Union)'s Smoking Cessation and Smoke-free Environments for TB Patients 2010 | in-person | the health-care provider | 2-3 min | No intervention | No intervention | the health-care provider |  | 6 months |
| Jackson 2004 |  | In-person , Printed Material | Male Research Assistant | Not reported |  | No intervention | Male Research Assistant | Not reported | 3- month follow up  6- month follow-up" |
| Jayakrishnan 2013 | multiple approaches- educational material, group & individual counselling | in-person/telephonic, printed materials | medical social workers | 6 months | Educational material | printed materials | medical social workers | - | 12 months |
| Jiang 2019 | Arm 1- Ask (screen for tobacco use), Advise to quit, Assess readiness to quit, and Assist (brief counselling and educational materials) (4As/ARM 1) to enhance implementation of TDT guidelines into routine care: This was informed by the US PHS guideline for Treating Tobacco Use and Dependence25 and the WHO Tobacco Control Package for Building Capacity for Tobacco Control in primary care | in-person | trained healthcare providers | Not reported | Arm 2- 4As plus a system for providers to refer smokers to a trained VHW for three sessions of in-person cessation counselling (4As+R/ARM) to enhance implementation of TDT guidelines into routine care: | In-person | village health workers (VHWs) | Not reported | 6 months |
| Koyun 2016 | transtheoretical model (TTM) was developed by Prochaska and DiClemente:  4 components- 1. stage of change (SOC: precontemplation, contemplation, preparation, action, and maintenance), which explains an individual’s thoughts and behaviours regarding how to change behaviour; 2. processes of change (POC), which explains what methods are used by the individual while changing behaviours; 3. self-efficacy (SE), which analyses the self-confidence of the individual regarding how long she/he will be able to resist the desire to smoke; 4. decisional balance (DB), which explains the pros and cons of change | in-person, printed materials | research staff | 6 months, counselling and training interviews about 45–60 min | Face to face interviews three times. | in-person, printed materials | research staff | 6 months | 1, 1.5, 2, and 6 months |
| Kumar 2017 | 5As approach recommended by the US Department of Health and Human Services for primary care clinicians/health care personnel: Ask the patient if he/she uses tobacco, advise him/her to quit, Assess willingness to make a quit attempt, Assist him/ her in making a quit attempt, and Arrange for fol- low-up contact to prevent relapse | in-person, printed materials | Physician and counsellor | Counselling- 15– 20 min | brochure containing smoking cessation information, and standard counselling | in-person, printed materials | counsellor | 15– 20 min | 1 month |
| Kumar 2020 | T1 -Bupropion SR along with standard counselling T2 -Enhanced counselling arm | In person, Printed material   In Person, Printed Material | T2: trained staff member | T2 - Not reported | standard- RNTCP guidelines modules to program managers 5 A approach (Ask, Advise, Assess, Assist and Arrange) and 5R approach (Relevance, Risk, Re- wards, Roadblocks, and Repetition). | In-person | Medical Officer | once | at 2nd and 6th month |
| Liao 2018 | motivational messages and behaviour change techniques- - encouraged participants to persevere with the quit attempt and focus on their success - positive feedback and emphasized the benefits achieved by quitting, as well as providing information about the consequences of smoking, how to quit and remain abstinent, and how others would approve of successful abstinence.  - The behaviour changing messages prompted participants to get rid of cigarettes, ashtrays, and lighters and to avoid environments where they would normally smoke, and encouraged participants to identify the challenges of quitting and plan how to overcome them.  - information relevant to quitting—e.g., symptoms to expect on quitting, tips to cope with craving, tips to avoid weight gain and improve nutrition, advice on avoiding smoking triggers, instructions on breathing exercises to perform instead of smoking, and motivational support and distraction | mobile messaging | Not reported | 24 weeks | No intervention messages, Happy Quit program booklet after completion of the trial. | mobile messaging | Not reported | 24 weeks | 1, 4, 8, 12, 16, 20, and 24 weeks |
| Loke 2005 | standardised advice, educational booklet | in-person, printed materials | Obstetrician | Initial advice session took about 2–3 min, and each reminder took about 1 and half min | No intervention | No intervention | No intervention | - | 3 to 5 months |
| Lou 2013 | brief smoking cessation advice | In-Person | Trained Family Physicians | Once a week at the first month  Once a month until the end the study | Usual Care | In-person | Healthcare providers | Not reported | Every six months in the follow-up period for two years. |
| Louwagie 2014 | Brief MI, and Smoking cessation booklet | in-person, printed materials | Trained LHCW | 15–20 minutes | short standardized smoking cessation message, and smoking cessation booklet supplied by the National Council against Smoking of South Africa | in-person, printed materials | Trained Nurse | once | "1-, 3- and 6-month TB treatment visits." |
| Luo 2018 | intensive personalized intervention using ‘5As+5Rs’ (IPANR)- counselling during hospitalization, telephone counselling and self-help materials (such as the book called “The easy way to stop smoking”) | In-person, telephonic and printed materials | cardiologists with extensive experience in smoking cessation | 6 months,  first session: 30–45 min;  second and third session: 10–30 min | The motivational interventions based on PHS 5Rs | in-person, telephonic and printed materials | Trained cardiologists | 6 months, each session 10-15 minutes | "Assessments were conducted at weeks 9, 12, and 24 |
| Mali, 2017 | A stop smokeless and smoking tobacco in school trial: A Stop Smoking in School Trial (ASSIST)-like peer-led intervention | in-person | peer-supporters from the same schools and classes | 6 months | No intervention | No intervention | No intervention | - | after 6 months and 1 year |
| Mehdi 2020 | Pharmacotherapy | in-person | Specialist | 2 weeks | Combined Pharmacotherapy + Psychotherapy | In-person | Specialist | 1st session, 45 Min | 2,4,6,8 and 12 weeks |
| Mishra 2010 | Arm 2: Active Health Education (HE) sessions followed by focus group discussion (FGD)  Arm 3 : Active HE sessions followed by FGD and Behavioural Therapy (BT) in the form of one-to-one counselling  Arm 4 : Active HE sessions were followed by FGD, BT, and Pharmacotherapy (PT). | Presentations, FDGs,            Counselling   Pharmacotherapy | Expert tobacco counsellor | 12 months | Arm 1: Health Education Pamphlets | printed materials | - | once | Follow-up was conducted intervals of 2-3 months |
| Naik 2014 | Motivational intervention | in-person | Not reported | Not reported | Not reported | Not reported | Not reported | Not reported | 6 months |
| Rajanandh 2012 | motivation enhancement counselling | in-person, printed materials | Not reported | 3 months,  each session- 10 minutes | Usual care | Not reported | Not reported | Not reported | 3 months |
| Sarkar 2017 | tobacco quit advice, and short training in yogic breathing exercises. | In-person | Trained research team | 15 min | Usual care- single control session involving very brief quit advice | In-person | Trained research team | 1 min | 4 weeks and 7 months |
| Siddiqi 2013 | Behavioural support intervention        Free 7-week course of bupropion in addition to BSS | In-person, printed materials       In-person, Pharmacotherapy | DOT facilitators        DOT facilitators, physicians | 1st session- 30-minutes, second session- 10 minutes.  7-week course of bupropion | usual care and the self-help leaflet | In-person | DOT facilitators | once | "BSS + and BSS groups- 1, 5 and 25 weeks  control group- 5 and 25 weeks." |
| Wu 2017 | Smoking-reduction intervention (SRI)- very brief advice for about one minute | In-person and telephonic counselling | Physicians and medical students, trained counsellors | 12 months, each session- 1 minute | Exercise- and diet-advice (EDA) control group | in-person, telephonic | Physicians and medical students, trained counsellors | 12 months, each session- 1 minute | 12 months |
| Xia 2020 | Video: Video content was developed using the theory of planned behaviour.  Text message intervention | In-Person | Research nurses | 1 video was sent to each participant in weeks 1, 3, 5, and 7. 1 text was sent to each participant in weeks 1, 3, 5, and 7. | Brief advice on smoking cessation and a leaflet published by the Chinese Center for Health Education | In-person | Research nurses | At baseline | Follow-up telephone calls were conducted at 1 week and 1, 3, and 6 months |
| Yahya 2018 | 5A’s group: five steps in the 5A’s intervention developed by Fiore et al. (2008) & 5R strategies | in-person, printed materials | Dental Public Health (DPH) specialists | 1 visit | Brief Advice | In-person | Dental Public Health (DPH) specialists | 1 visit, 1-5 min | 1, 3 & 6 months |
| Ybarra 2012 | telephone-based counselling approaches to smoking cessation, particularly those using CBT | mobile messaging | Research staff | 6 weeks | general quitting information | In-person | Research staff | once | 3 months |
| Yilmaz 2006 | Brief intervention emphasizing health risks of environmental tobacco smoke Arm 1: risks to children’s health were explained. Arm 2: risks of tobacco to the mother’s own health was discussed. | in-person, printed materials | Hospital nurse | 10 minutes | No smoking cessation advice. | In-person | Hospital nurse | - | 6 months |
| Yu 2017 | Counselling, Education Intervention        Educational Intervention, Text message intervention | In-person, Home visit, Printed Material       In-person, Home visit, Printed Material, Text message | Trained health care workers | Not reported | No intervention | No intervention | No intervention |  | Follow-up home visits were performed at 6 and 12 months |

## Supplementary Figure 1: Behavioural intervention versus active intervention. Outcome: Continuous abstinence at 6 months


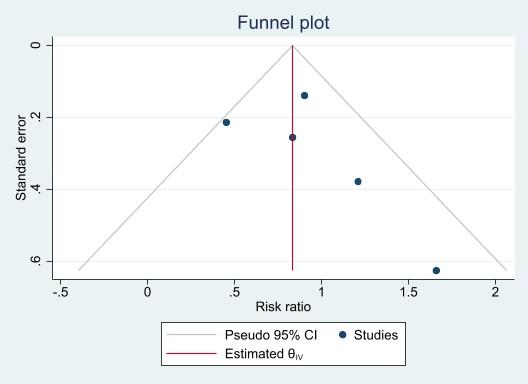


## Supplementary Figure 2: Behavioural intervention versus usual care. Outcome: Continuous abstinence at 6 months


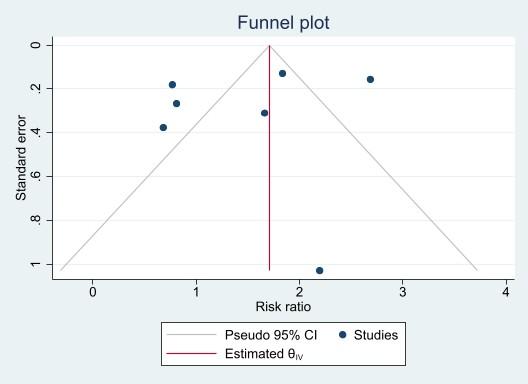


## Supplementary Figure 3: Behavioural intervention versus active intervention. Outcome: Point prevalence abstinence at 6 months


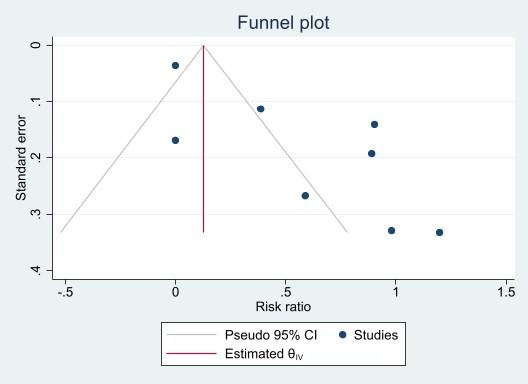


## Supplementary Figure 4: Behavioural intervention versus usual care. Outcome: Point prevalence abstinence at 6 months


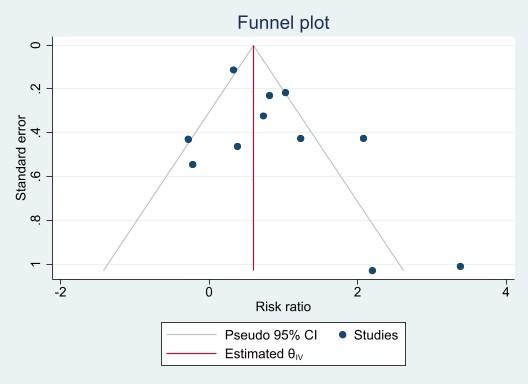


## **Supplementary Table 4: PRISMA 2020 Checklist**

| Section and Topic | Item # | Checklist item | Location where item is reported |
| --- | --- | --- | --- |
| TITLE | | |  |
| Title | 1 | Identify the report as a systematic review. | 1 |
| ABSTRACT | | |  |
| Abstract | 2 | See the PRISMA 2020 for Abstracts checklist. | 2 |
| INTRODUCTION | | |  |
| Rationale | 3 | Describe the rationale for the review in the context of existing knowledge. | 3-4 |
| Objectives | 4 | Provide an explicit statement of the objective(s) or question(s) the review addresses. | 4 |
| METHODS | | |  |
| Eligibility criteria | 5 | Specify the inclusion and exclusion criteria for the review and how studies were grouped for the syntheses. | 4 |
| Information sources | 6 | Specify all databases, registers, websites, organisations, reference lists and other sources searched or consulted to identify studies. Specify the date when each source was last searched or consulted. | 5 |
| Search strategy | 7 | Present the full search strategies for all databases, registers, and websites, including any filters and limits used. | Supplementary material 1 |
| Selection process | 8 | Specify the methods used to decide whether a study met the inclusion criteria of the review, including how many reviewers screened each record and each report retrieved, whether they worked independently, and if applicable, details of automation tools used in the process. | 5 |
| Data collection process | 9 | Specify the methods used to collect data from reports, including how many reviewers collected data from each report, whether they worked independently, any processes for obtaining or confirming data from study investigators, and if applicable, details of automation tools used in the process. | 5 |
| Data items | 10a | List and define all outcomes for which data were sought. Specify whether all results that were compatible with each outcome domain in each study were sought (e.g., for all measures, time points, analyses), and if not, the methods used to decide which results to collect. | 6 |
|  | 10b | List and define all other variables for which data were sought (e.g., participant and intervention characteristics, funding sources). Describe any assumptions made about any missing or unclear information. | N/A |
| Study risk of bias assessment | 11 | Specify the methods used to assess risk of bias in the included studies, including details of the tool(s) used, how many reviewers assessed each study and whether they worked independently, and if applicable, details of automation tools used in the process. | 5 |
| Effect measures | 12 | Specify for each outcome the effect measure(s) (e.g., risk ratio, mean difference) used in the synthesis or presentation of results. | 6 |
| Synthesis methods | 13a | Describe the processes used to decide which studies were eligible for each synthesis (e.g., tabulating the study intervention characteristics and comparing against the planned groups for each synthesis | 6 |
|  | 13b | Describe any methods required to prepare the data for presentation or synthesis, such as handling of missing summary statistics, or data conversions. | 6 |
|  | 13c | Describe any methods used to tabulate or visually display results of individual studies and syntheses. | 6 |
|  | 13d | Describe any methods used to synthesize results and provide a rationale for the choice(s). If meta-analysis was performed, describe the model(s), method(s) to identify the presence and extent of statistical heterogeneity, and software package(s) used. | 6 |
|  | 13e | Describe any methods used to explore possible causes of heterogeneity among study results (e.g., subgroup analysis, meta-regression). | 6 |
|  | 13f | Describe any sensitivity analyses conducted to assess robustness of the synthesized results. | 6 |
| Reporting bias assessment | 14 | Describe any methods used to assess risk of bias due to missing results in a synthesis (arising from reporting biases). | 6 |
| Certainty assessment | 15 | Describe any methods used to assess certainty (or confidence) in the body of evidence for an outcome. | 6 |
| RESULTS | | |  |
| Study selection | 16a | Describe the results of the search and selection process, from the number of records identified in the search to the number of studies included in the review, ideally using a flow diagram. | 7 |
|  | 16b | Cite studies that might appear to meet the inclusion criteria, but which were excluded, and explain why they were excluded. | N/A |
| Study characteristics | 17 | Cite each included study and present its characteristics. | 8-12 |
| Risk of bias in studies | 18 | Present assessments of risk of bias for each included study. | 21 |
| Results of individual studies | 19 | For all outcomes, present, for each study: (a) summary statistics for each group (where appropriate) and (b) an effect estimate and its precision (e.g. confidence/credible interval), ideally using structured tables or plots. | 13- 20, Supplementary Table 1 |
| Results of syntheses | 20a | For each synthesis, briefly summarise the characteristics and risk of bias among contributing studies. | 22 |
|  | 20b | Present results of all statistical syntheses conducted. If meta-analysis was done, present for each the summary estimate and its precision (e.g. confidence/credible interval) and measures of statistical heterogeneity. If comparing groups, describe the direction of the effect. | 22-24 |
|  | 20c | Present results of all investigations of possible causes of heterogeneity among study results. | 22-24 |
|  | 20d | Present results of all sensitivity analyses conducted to assess the robustness of the synthesized results. | 24 |
| Reporting biases | 21 | Present assessments of risk of bias due to missing results (arising from reporting biases) for each synthesis assessed. | N/A |
| Certainty of evidence | 22 | Present assessments of certainty (or confidence) in the body of evidence for each outcome assessed. | 25-26 |
| DISCUSSION | | |  |
| Discussion | 23a | Provide a general interpretation of the results in the context of other evidence. | 27 |
|  | 23b | Discuss any limitations of the evidence included in the review. | 27 |
|  | 23c | Discuss any limitations of the review processes used. | 28 |
|  | 23d | Discuss implications of the results for practice, policy, and future research. | 28 |
| OTHER INFORMATION | | |  |
| Registration and protocol | 24a | Provide registration information for the review, including register name and registration number, or state that the review was not registered. | 4 |
|  | 24b | Indicate where the review protocol can be accessed, or state that a protocol was not prepared. | 4 |
|  | 24c | Describe and explain any amendments to information provided at registration or in the protocol. | 4 |
| Support | 25 | Describe sources of financial or non-financial support for the review, and the role of the funders or sponsors in the review. | 28 |
| Competing interests | 26 | Declare any competing interests of review authors. | 28 |
| Availability of data, code, and other materials | 27 | Report which of the following are publicly available and where they can be found: template data collection forms; data extracted from included studies; data used for all analyses; analytic code; any other materials used in the review. | 29 |

## Supplementary material 1: Search strategy

**Search Strategy for MedLine:**

1. Tobacco.tw
2. Smoker*.tw
3. Smoking.tw
4. Cigarette*.tw
5. Smokeless tobacco.tw
6. Smok* tobacco.tw
7. Chewing tobacco.tw
8. Chewed tobacco.tw
9. Beedi.tw
10. Paan.tw
11. Gutk?a.tw
12. Chew.tw
13. Snuff*.tw
14. Hooka*.tw
15. Cigar*.tw
16. Betel*.tw
17. Areca*.tw
18. **OR (1-17)**
19. Tobacco use disorder/
20. Smokers/
21. Smoking/
22. Tobacco products/
23. Smokeless tobacco/
24. **OR (19-23)**
25. **18 or 24**
26. Cessation.tw
27. Psychotherap*.tw
28. Therap*.tw
29. Counsel?ing.tw
30. Psychosocial intervention*.tw
31. Psychosocial treatment*.tw
32. Psychosocial therap*.tw
33. Psychosocial support.tw
34. Psychological intervention*.tw
35. Psychological treatment*.tw
36. Psychological therap*.tw
37. Psychological support.tw
38. Coping.tw
39. Support*.tw
40. Intervention*.tw
41. Treatment*.tw
42. Program*.tw
43. Package*.tw
44. Counsel*ing.tw
45. Therap*.tw
46. Behavioural intervention.tw
47. Self-reported abstinence.tw
48. Prompts reminders.tw
49. Praise.tw
50. Action planning.tw
51. Behaviour substitution.tw
52. Goal setting.tw
53. Behaviour feedback.tw
54. Habit reversal.tw
55. Self-monitoring.tw
56. **OR (26-55)**
57. Psychotherapy/
58. Therapy/
59. Counselling/
60. Psychosocial intervention/
61. Psychosocial treatment/
62. Psychosocial therapy/
63. Psychosocial support/
64. Psychological intervention/
65. Psychological treatment/
66. Psychological therapy/
67. Psychological support/
68. Coping/
69. Support/
70. **OR (57-69)**
71. **56 OR 70**
72. Developing.tw
73. Less$ developed.tw
74. Under developed.tw
75. Underdeveloped.tw
76. middle income.tw
77. low income.tw
78. lower income.tw
79. third world.tw
80. transitional.tw
81. **OR (72-80)**
82. countr$.tw
83. nation$.tw
84. population$.tw
85. world.tw
86. **OR (82-85)**
87. **81 AND 86**
88. L?MIC.tw
89. L?MICs.tw
90. **OR (88-89)**
91. Afghanistan.tw
92. Albania.tw
93. Algeria.tw
94. Angola.tw
95. Antigua.tw
96. Barbuda.tw
97. Argentina.tw
98. Armenia$.tw
99. Aruba.tw
100. Azerbaijan.tw
101. Bangladesh.tw
102. Benin.tw
103. Byelarus$.tw
104. Belarus.tw
105. Belorussian.tw
106. Belorussia.tw
107. Belize.tw
108. Bhutan.tw
109. Bolivia.tw
110. Bosnia.tw
111. Herzegovina.tw
112. Hercegovina.tw
113. Botswana.tw
114. Brazil.tw
115. Bulgaria.tw
116. Burkina Faso.tw
117. Burkina Fasso.tw
118. Upper Volta.tw
119. Burundi.tw
120. Urundi.tw
121. Cambodia.tw
122. Khmer Republic.tw
123. Kampuchea.tw
124. Cameroon$.tw
125. Cameron$.tw
126. Cape Verde.tw
127. Central African Republic.tw
128. Chad.tw
129. Chile.tw
130. China.tw
131. Colombia.tw
132. Comoros.tw
133. Comoro Islands.tw
134. Comores.tw
135. Mayotte.tw
136. Congo.tw
137. Zaire.tw
138. Costa Rica.tw
139. Cote d Ivoire.tw
140. Ivory Coast.tw
141. Croatia.tw
142. Cuba.tw
143. Cyprus.tw
144. Czechoslovakia.tw
145. Czech Republic.tw
146. Slovak$.tw
147. Djibouti.tw
148. French Somaliland.tw
149. Dominica$.tw
150. East Timor.tw
151. East Timur.tw
152. Timor Leste.tw
153. Ecuador.tw
154. Egypt.tw
155. El Salvador.tw
156. Eritrea.tw
157. Estonia.tw
158. Ethiopia.tw
159. Fiji.tw
160. Gabon$.tw
161. Gambia.tw
162. Gaza.tw
163. Georgia$ Republic.tw
164. Ghana.tw
165. Gold Coast.tw
166. Grenada.tw
167. Guatemala.tw
168. Guinea.tw
169. Guam.tw
170. Guiana.tw
171. Guyana.tw
172. Haiti.tw
173. Honduras.tw
174. India.tw
175. Maldives.tw
176. Indonesia.tw
177. Iran.tw
178. Iraq.tw
179. Jamaica.tw
180. Jordan.tw
181. Kazakh$.tw
182. Kenya.tw
183. Kiribati.tw
184. Korea.tw
185. Kosovo.tw
186. Kyrgyz$.tw
187. Kirghiz$.tw
188. Kirgizstan.tw
189. Lao PDR.tw
190. Laos.tw
191. Latvia.tw
192. Lebanon.tw
193. Lesotho.tw
194. Basutoland.tw
195. Liberia.tw
196. Libya.tw
197. Lithuania.tw
198. Macedonia.tw
199. Madagasca$.tw
200. Malagasy.tw
201. Malay$.tw
202. Sabah.tw
203. Sarawak.tw
204. Malawi.tw
205. Nyasaland.tw
206. Mali.tw
207. Marshall Islands.tw
208. Mauritania.tw
209. Mauritius.tw
210. Agalega Islands.tw
211. Mexico.tw
212. Micronesia.tw
213. Middle East.tw
214. Moldov$.tw
215. Mongolia.tw
216. Montenegro.tw
217. Morocco.tw
218. Ifni.tw
219. Mozambique.tw
220. Myanma$.tw
221. Burma.tw
222. Namibia.tw
223. Nepal.tw
224. Netherlands.tw
225. Antilles.tw
226. New Caledonia.tw
227. Nicaragua.tw
228. Niger$.tw
229. Mariana Islands.tw
230. Oman.tw
231. Muscat.tw
232. Pakistan.tw
233. Palau.tw
234. Palestine.tw
235. Panama.tw
236. Paraguay.tw
237. Peru.tw
238. Philippines.tw
239. Philipines.tw
240. Phillipines.tw
241. Phillippines.tw
242. Romania.tw
243. Rumania.tw
244. Roumania.tw
245. Russia£.tw
246. Rwanda.tw
247. Ruanda.tw
248. Saint Kitts.tw
249. St Kitts.tw
250. Nevis.tw
251. Saint Lucia.tw
252. St Lucia.tw
253. Saint Vincent.tw
254. St Vincent.tw
255. Grenadines.tw
256. Samoa$.tw
257. Islands or Navigator Island.tw
258. Navigator Islands.tw
259. Sao Tome.tw
260. Senegal.tw
261. Serbia.tw
262. Montenegro.tw
263. Seychelles.tw
264. Sierra Leone.tw
265. Slovenia.tw
266. Sri Lanka.tw
267. Ceylon.tw
268. Solomon Islands.tw
269. Somali$.tw
270. Sudan.tw
271. Surinam$.tw
272. Swaziland.tw
273. Syria.tw
274. Tajikistan.tw
275. Tadzhikistan.tw
276. Tadjikistan.tw
277. Tadzhik.tw
278. Tanzania.tw
279. Thailand.tw
280. Togo$.tw
281. Tonga.tw
282. Trinidad.tw
283. Tobago.tw
284. Tunisia.tw
285. Turkey.tw
286. Turkmen$.tw
287. Uganda.tw
288. Ukraine.tw
289. Uruguay.tw
290. USSR.tw
291. Soviet Union.tw
292. Union of Soviet Socialist Republics.tw
293. Uzbek$.tw
294. Vanuatu.tw
295. New Hebrides.tw
296. Venezuela.tw
297. Vietnam.tw
298. Viet Nam.tw
299. West Bank.tw
300. Yemen.tw
301. Yugoslavia.tw
302. Zambia.tw
303. Zimbabwe.tw
304. Rhodesia.tw
305. **OR (91-304)**
306. **87 OR 90 OR 305**
307. Randomi?ed Controlled Trial.tw
308. RCT.tw
309. Trial.tw
310. Clinical [trial.tw](http://trial.tw)
311. Controlled Clinical Trial.tw
312. Pragmatic Clinical Trial.tw
313. Equivalence Trial.tw
314. Adaptive Clinical Trial.tw
315. Cluster randomi?ed controlled trial.tw
316. **OR (307-315)**
317. Clinical Trial/
318. Random-Allocation/
319. randomized-controlled trials/
320. randomised-controlled trials/
321. double-blind-method/
322. single-blind-method/
323. Follow-Up-Studies/
324. Evaluation-Studies/
325. **OR (317-324)**
326. **316 OR 325**
327. **25 AND 71 AND 306 AND 326**

- Search strategy above was run on Medline, PsycInfo, Embase and Global Health (using the OVID platform) and CINAHL.
- For Cochrane Central, the search strategy above was run without the search terms for the ‘trials’ concept
- Other search strategies will be based on this and revised as appropriate to meet the requirements of the particular database.
- For AJOL we used the term ‘tobacco cessation’
- For LILACS we used ‘tobacco’ as a search term and then used the ‘controlled clinical trial’ filter
